# Fear-Specific Technophobia as a Mediator Between Daily Technical Support and Instrumental Versus Hedonic Technology Use in Later Life: Cross-Sectional Survey Study

**DOI:** 10.2196/93063

**Published:** 2026-10-23

**Authors:** Yu Yang, Shuo Dai, Siyu Yin, Wanyu Xi, Qi Li

**Affiliations:** 1 Hohai University, Nanjing, Jiangsu, China; 2 Jiangsu Open University, Nanjing, Jiangsu, China; 3 School of Aging Services and Management, Nanjing University of Chinese Medicine, No.138 Xianlin Road, Nanjing, Jiangsu, 210023, China, 86 15850683105; 4 School of Business Administration (MBA), Key Research Institute of Humanities and Social Sciences at Universities Modern Business Research Center, Zhejiang Gongshang University, Hangzhou, Zhejiang, China

**Keywords:** technophobia, daily technical support, technology adoption, older adults, digital inclusion

## Abstract

**Background:**

Population aging and rapid digitalization have intensified concerns about older adults’ digital participation. Although access disparities have narrowed, older adults continue to differ from younger groups in how they use digital technologies, with their use skewed toward instrumental rather than hedonic purposes. Technophobia is a salient psychological barrier; yet, its distinct fear dimensions and their links to different forms of technology use remain insufficiently differentiated. Daily technical support (DTS) may provide an important sociotechnical context for understanding these relationships.

**Objective:**

This study aimed to test how DTS promotes older adults’ instrumental and hedonic technology use by reducing multidimensional technophobia (fear of inconvenience, fear of personal failure, and human-machine ambiguity) and whether these mechanisms differ by gender.

**Methods:**

A cross-sectional survey was conducted among Chinese adults aged ≥55 years recruited via online platforms and offline communities in Nanjing (June-October 2024). A total of 212 valid responses were included (mean age 66.96, SD 6.96, range 55‐86 years; 142/212, 68.4% women). Measures assessed day-to-day technical support, 3 dimensions of technophobia, instrumental versus hedonic technology use, and demographic variables. We used partial least squares structural equation modeling (PLS-SEM) with 5000 bootstrap resamples to test direct, indirect, and moderated mediation effects, reporting 95% bias-corrected and accelerated (BCa) CI.

**Results:**

DTS significantly reduced fear of inconvenience (β=−0.455, 95% BCa CI −0.632 to −0.229; *P*<.001) but did not significantly affect fear of personal failure (*P*=.12) or human-machine ambiguity (*P*=.26). Fear of inconvenience and human-machine ambiguity negatively predicted instrumental use (IU; β=−0.215, 95% BCa CI −0.410 to −0.009; *P*=.05 and β=−0.211, 95% BCa CI −0.389 to −0.001; *P*=.04). DTS had a positive direct effect on IU (β=0.104, 95% BCa CI 0.009 to 0.203; *P*=.04) but not on hedonic use (HU; β=0.075; *P*=.11). The indirect effect of DTS on IU through fear of inconvenience was significant (β=0.097, 95% BCa CI 0.020-0.204). Gender moderated the effect of DTS on fear of inconvenience (β=0.287, 95% BCa CI 0.052-0.518; *P*=.02), and such conditional indirect effect was significant for men (β=0.098, 95% BCa CI 0.001-0.219) but not for women (β=0.036, 95% BCa CI −0.002 to 0.116).

**Conclusions:**

DTS was associated with greater instrumental technology use among older adults through lower fear of inconvenience, with a stronger indirect association among men, whereas no comparable pathway emerged for HU. This suggests that gender may shape how older adults benefit psychologically from technical support rather than reflect general differences in support needs. Interventions should move beyond one-size-fits-all approaches by providing targeted, task-oriented support that addresses older adults’ specific technological fears and practical barriers.

## Introduction

### Background

Population aging is accelerating worldwide, with the proportion of people aged 60 years and older projected to nearly double from 12% in 2015 to 22% by 2050 [1]. Meanwhile, with the rapid development of information and communication technologies (ICTs), digital technologies have become increasingly embedded in older adults’ everyday lives, mediating routine activities such as banking, shopping, health management, and social interaction and making digital competence increasingly comparable to foundational literacy [2,3]. The first-level digital divide, which refers to inequalities in access to digital technologies [4], has narrowed substantially with the widespread diffusion of internet-enabled devices and improved connectivity [5-7]. In China, for example, the number of internet users aged 60 years and older increased from 119 million in December 2021 to approximately 173 million in December 2025, an increase of approximately 45.4% over 4 years [8,9]. Nevertheless, older adults remain disproportionately disadvantaged in digital access, with an internet penetration rate of 53.7%, compared with 80.1% for the population as a whole [8]. In addition, despite the narrowing first-level digital divide, substantial inequalities persist at the second and third levels, reflected in disparities in how older adults use digital technologies and in the digital skills and competencies needed for effective and meaningful engagement [10,11]. Indeed, older adults’ online engagement remains markedly skewed toward instrumental uses (IUs; eg, online services and communication) rather than hedonic uses (HUs; eg, entertainment, gaming, and social media) [11-13]. For instance, in the United Kingdom, 58% of older internet users (ages 65‐74 years) shop online and 65% bank online, whereas only about 32% play online games [14].

Barriers to ICT adoption among older adults are multifaceted, encompassing health-related limitations, inadequate digital skills, resource constraints such as cost and connectivity, insufficient support networks, and psychological factors [15]. While many of these barriers primarily limit older adults’ opportunities or capacity to engage with technology, psychological factors may exert a more immediate influence on adoption decisions by shaping how older adults perceive their technological capabilities, evaluate the benefits and demands of technology, and respond to difficulties encountered during use. Accordingly, psychological barriers—particularly technophobia—are salient for older adults. Technophobia is commonly defined as a fear or anxiety about technology, and it is associated with delayed or hesitant adoption, especially among individuals with limited digital literacy or inaccurate beliefs about technology [11,16]. Although scholars have called for conceptualizing technophobia as multidimensional, much of the literature continues to treat it as a unidimensional construct, limiting fine-grained insight into how distinct fears map onto different forms of technology use [16-19]. Moreover, while prior research has examined demographic correlates of technophobia and shown that structured interventions (eg, training and intergenerational mentoring) can alleviate it [20,21], evidence remains scarce on whether and how spontaneous day-to-day technical support—more typical of naturalistic settings—shapes technophobia within a broader sociotechnical context.

To address these gaps, this study examines how daily technical support (DTS) influences older adults’ instrumental versus hedonic technology use through multiple fear-related dimensions of technophobia. We further test whether these indirect effects vary by gender, given documented gender differences in support-seeking motivations and technology-related stereotypes [22]. By clarifying which dimensions of technophobia are most responsive to everyday support—and for whom—this research advances digital divide scholarship and informs more targeted, age-friendly support and training strategies.

### Literature Review and Hypotheses Development

#### The Multidimensionality of Technophobia

Originated from the concept of computer anxiety, technophobia was initially conceptualized as a unidimensional construct capturing negative psychological responses to technology in general [23,24]. This early view closely resembles technology anxiety or digital anxiety, which refers primarily to restlessness or apprehensive tension when one has to use or learn a new technology [25]. Subsequent research has refined this perspective, arguing that technophobia is not merely a simple emotional reaction (ie, anxiety); rather, technology anxiety constitutes only 1 facet of technophobia [17,26]. Accordingly, technophobia is better understood as an umbrella, higher-order phenomenon comprising multiple dimensions that reflect the cognitive appraisals and beliefs underlying the sources of fear during technology adoption.

Some studies have approached technophobia from an affective perspective, conceptualizing it primarily in terms of anxiety, discomfort, or other negative emotional responses to technology [27-29], with measures including techno-anxiety, techno-paranoia, and privacy concerns [26]. A different line of research has focused on the specific sources from which such fear arises. Sinkovics et al (2002) [19] proposed 3 dimensions of technophobia from the perspective of the underlying sources of fear; it distinguishes self-related, internal sources of fear from externally situated concerns embedded in the usage context. Fear of personal failure refers to the anticipated threat to one’s competence and self-worth when engaging with technology—specifically, the concern that one may be unable to use it correctly, may make errors, or may be perceived as incapable. Fear of inconvenience reflects a more instrumental concern about whether the effort required to use a technology is justified by its anticipated benefits. Human-machine ambiguity captures discomfort rooted in one’s preferences and beliefs about everyday action and interpersonal exchange, particularly when human contact is substituted by machine-mediated interaction. Subsequent research has further reinforced the view of technophobia as a multidimensional construct. For example, Khasawneh (2018) [17] identified 5 dimensions of technophobia, including techno-anxiety, techno-paranoia, techno-fear, techno-cybernetic revolt, and communication device avoidance. Although more differentiated, broadly capture fear sources similar to those represented in Sinkovics et al [19] 3D framework.

Prior empirical work has consistently shown that older adults tend to report higher levels of technophobia than younger cohorts [16,18]. Technophobia has also been associated with avoidance and reduced technology engagement, such that older adults may use digital technologies less frequently, limit themselves to a narrower range of applications, and show less willingness to adopt unfamiliar technologies [30]. However, much of the existing literature has treated technophobia as a global or unidimensional construct, providing limited insight into which specific sources of fear are associated with particular technology-use behaviors. By differentiating among distinct fear sources, Sinkovics et al [19] framework offers a more fine-grained basis for identifying the psychological barriers underlying older adults’ technology use and for considering forms of support that are better matched to those barriers. Accordingly, the current study adopts this 3D, fear-specific framework to examine how different forms of technophobia are associated with older adults’ technology use.

#### The Instrumental Versus HU of Technology

Scholars of information systems commonly distinguish 2 types of IT according to their primary purposes and functional orientations, utilitarian (instrumental) systems that support productivity and task accomplishment, and hedonic systems that foreground enjoyment, entertainment, and self-expression [31,32]. This typology draws on the hedonic consumption tradition that differentiates utilitarian from hedonic products and experiences [33]. It underscores that information systems are embedded not only in work or education settings for job and study-related activities, but also in domestic and leisure contexts where technology use itself can provide enjoyment and experiential value. Correspondingly, users’ purposes for daily technology use tend to cluster into 2 broad motivation-driven categories: instrumental purposes (eg, banking, information search, and health management) and hedonic purposes (eg, streaming, gaming, and social media). Although some digital technologies can serve both instrumental and hedonic purposes, this dichotomy remains analytically useful because the psychological beliefs underlying technology acceptance may differ depending on the primary purpose for which a technology is used.

The Technology Acceptance Model (TAM) provides the primary theoretical foundation for understanding why individuals adopt and use technology. TAM proposes that technology acceptance is largely determined by 2 key beliefs: perceived usefulness and perceived ease of use [32,34]. These 2 beliefs reflect different motivational considerations underlying technology use. Perceived usefulness is closely associated with extrinsic motivation because users adopt a technology in anticipation of instrumental outcomes, such as improved performance, greater efficiency, or task accomplishment [35]. Perceived ease of use, in contrast, reflects the effort required to interact with a technology and is more closely related to the experiential process of technology use; by reducing effort and facilitating a more fluent interaction, ease of use can support intrinsically motivated experiences such as enjoyment and playfulness [36]. Importantly, the relative importance of these motivational mechanisms varies across technology-use contexts. In instrumental contexts, technology is primarily used as a means to accomplish an external goal. Consequently, extrinsic motivation is particularly salient, and perceived usefulness becomes a central determinant of adoption because users evaluate whether the technology helps them perform tasks more effectively or efficiently. In hedonic contexts, by contrast, technology use is more strongly driven by intrinsic rewards derived from the use experience itself, such as enjoyment, pleasure, playfulness, and immersion. Accordingly, perceived enjoyment becomes particularly important, while ease of use facilitates hedonic motivation by reducing interactional effort and allowing users to experience these intrinsic rewards more readily [32,35]. Thus, from a TAM perspective, the distinction between instrumental and hedonic technology use reflects not merely differences in technological functions, but differences in the motivational beliefs that become most salient across use contexts.

#### The Impact of Technophobia on Different Use of Technology Among Older Adults

Building on TAM, which suggests that different technology-use contexts may be driven by different motivational beliefs, we further draw on the multidimensional view of technophobia proposed by Sinkovics et al [19] to examine how distinct sources of technological fear may map onto extrinsic versus intrinsic motivational orientations toward technology use [37,38]. In particular, fear of inconvenience aligns with extrinsic motivation, such that perceived usefulness from the TAM represents rational skepticism about the effort-benefit balance of using technology [35]. Older adults with a heightened fear of inconvenience perceive technology as overly burdensome, thereby reducing perceived value and discouraging adoption. Conversely, fear of personal failure relates closely to perceived ease of use and self-efficacy, which echoes the intrinsic motivation in TAM, where low self-confidence leads to anxiety about technological mistakes or inadequacies [34,39]. Older adults with a heightened fear of personal failure avoid technologies that require adaptation or learning, thus reinforcing digital exclusion [6,18]. In Sinkovics et al [19], the human-machine ambiguity dimension captures deep-seated preferences and beliefs about everyday action, particularly individuals’ comfort with human interaction when the “human element” is replaced by a machine. Recent research suggests that such substitution can undermine perceived autonomy, thereby threatening intrinsic motivation [40-42].

We build on the foregoing review to argue that the fear of inconvenience, which is a rational, extrinsic, effort-benefit appraisal, should most strongly depress iIU, where perceived usefulness is decisive. By contrast, fear of personal failure, as well as human-machine ambiguity, which erodes competence beliefs and undermines intrinsic engagement, should strongly curb HU, where perceived enjoyment and comfort are pivotal. This distinction motivates our domain-specific expectation that different technophobia dimensions map onto different adoption purposes rather than exerting uniform effects across all forms of technology use. Therefore, we propose the hypotheses mentioned below.

#### Hypothesis 1

Specific dimensions of technophobia will be negatively associated with older adults’ technology use, with different fear dimensions expected to be more relevant to different types of technology use. Specifically, (1) fear of inconvenience will be negatively associated with instrumental technology use, (2) fear of personal failure will be negatively associated with hedonic technology use, and (3) human-machine ambiguity will be negatively associated with hedonic technology use.

#### Technical Support and Its Positive Effects

In gerontological research, social support is widely recognized as a key social determinant of healthy aging, and it generally refers to the resources and assistance provided through social relationships, which help individuals feel cared for, valued, and part of a mutually obliging network [43]. It typically distinguishes between structural and functional dimensions of support. The structural aspect refers to the existence and quantity of social relationships, such as living arrangements, network size, and frequency of social interaction [44]. The functional aspect refers to the specific supportive functions that relationships serve, and 2 primary functional forms are studied. Emotional support involves empathy, caring, love, and someone to confide in during stress. Instrumental support, which is usually tangible, refers to practical help or services, such as assistance with daily tasks or financial aid [45]. A robust body of evidence suggests that a strong social support network of any functions is associated with better outcomes in late life, including improved health and longevity [46], better mental health (eg, reduced stress, loneliness, and depression) [47,48], and greater well-being (eg, life satisfaction and overall quality of life) [49,50].

Technical support, which often provides immediate resolution and encourages continued technology use, is primarily recognized as instrumental support. Such support can be structured through formal workshops, training sessions, or digital learning programs led by instructors; it can also be informal and embedded in daily life through ad-hoc help from peers, family, or volunteers [11,51]. Previous research has consistently demonstrated that technical support is a powerful facilitator for new technology adoption among older adults because it enables the immediate resolution of technical issues, thus underscoring the general importance of assistance in overcoming initial adoption hurdles [11,52]. Therefore, we propose hypothesis 2.

#### Hypothesis 2

DTS will promote the instrumental and HUs of technologies among older adults.

On the other hand, it is also found that daily family support that focuses on digital tasks helps to reduce older users’ overall digital anxiety [25]. A set of intervention studies demonstrate that technical support from internet training programs effectively reduced older adults’ technology anxiety while improving confidence [53], and similar improvements have been observed in collaborative peer-learning contexts [54]. With this basis, we argue that ongoing DTS can positively influence all 3 dimensions of technophobia. First, practical assistance alleviates the fear of inconvenience by simplifying the learning process and demonstrating that technology can make life easier [55]. Second, frequent support helps counter the fear of personal failure—knowing that mistakes can be fixed with help boosts self-efficacy through repeated successful experiences. Older adults particularly prefer assistance from trusted family or friends, as this reduces embarrassment and enhances comfort [56]. Third, having a human intermediary eases human-technology ambiguity, transforming a solitary, intimidating process into an interactive, supportive experience [57]. Therefore, we further proposed hypothesis 3 mentioned below.

#### Hypothesis 3

DTS will mitigate each dimension of technophobia among older adults, including (1) fear of inconvenience, (2) fear of personal failure, and (3) fear of human-machine ambiguity.

#### The Mediation Effect of Technophobia

While the direct positive impact of technical support on both perception and behavioral reaction toward technologies is well documented, research also suggested that this effect is often indirect and operates through users’ perceptions of the technology. As articulated in the TAM, external factors (such as training or support available in one’s environment) do not directly cause someone to adopt a technology; instead, they influence key perceptual drivers of adoption, such as the technology’s perceived ease of use and perceived usefulness [39,58]. For instance, workplace studies have found that when support is available, employees report high ease-of-use and usefulness beliefs about new IT systems, which then predict great adoption and usage [39]. Therefore, we further proposed the mediation effect of technophobia (hypothesis 4).

#### Hypothesis 4

Each dimension of technophobia will mediate the effect of DTS on both HU and IU of technologies. Specifically, (1) fear of inconvenience, (2) fear of personal failure, and (3) human-technology ambiguity will mediate the effect of DTS on both IU and HU of technologies among older adults.

#### The Moderation Effect of Gender

Social role theory posits that societally prescribed gender roles shape help-seeking tendencies and coping behaviors [59,60]. Across cultural contexts, masculinity norms emphasize competence, making help-seeking or admitting difficulty potentially threatening to a “competent man” identity; consequently, men are often more reluctant to seek assistance and report higher self-efficacy and lower anxiety than women [61]. Men’s achievement- and task-oriented roles further heighten their focus on performance payoffs and instrumental attributes [59]. In contrast, women are frequently stereotyped as less competent in masculine-typed domains such as technology and with comparatively lower self-efficacy may place greater value on environmental and interpersonal support to reduce anxiety and maintain positive self-perceptions [62]. Supporting evidence further showed that men’s technology adoption is more strongly driven by extrinsic motivation (perceived usefulness), whereas women are more responsive to perceived ease of use and internalized social norms [63,64].

On this basis, older adults, who were often socialized under more traditional gender norms, may differ in how they respond to DTS [22]. Because older men may view seeking help from others as a threat to their competence and treat support mainly as an instrumental fix, DTS may be more likely to reduce extrinsic, cost-related fears such as fear of inconvenience, but less likely to alleviate intrinsic, self-efficacy related fears such as fear of personal failure [65]. By contrast, older women may be more inclined to rely on social support when coping with difficult tasks; such support can reduce anxiety and bolster confidence as learners [66]. Thus, DTS may more effectively address women’s fear of personal failure. Finally, because fear of human-machine ambiguity reflects not only the interaction experience but also relational beliefs in everyday activities, and because women tend to be more attuned to internal feelings and social norms, technical support may more strongly reduce this fear among older women than older men. In sum, we propose hypothesis 5 as mentioned below.

#### Hypothesis 5

Gender moderates the effect of DTS on technophobia. Specifically, (1) technical support will likely reduce the fear of inconvenience among older men (vs women). By contrast, (2) technical support will likely reduce the fear of personal failure, and (3) the fear of human-machine ambiguity among older women (vs men).

Figure 1 shows the overall conceptual model of this research.

**Figure 1.**
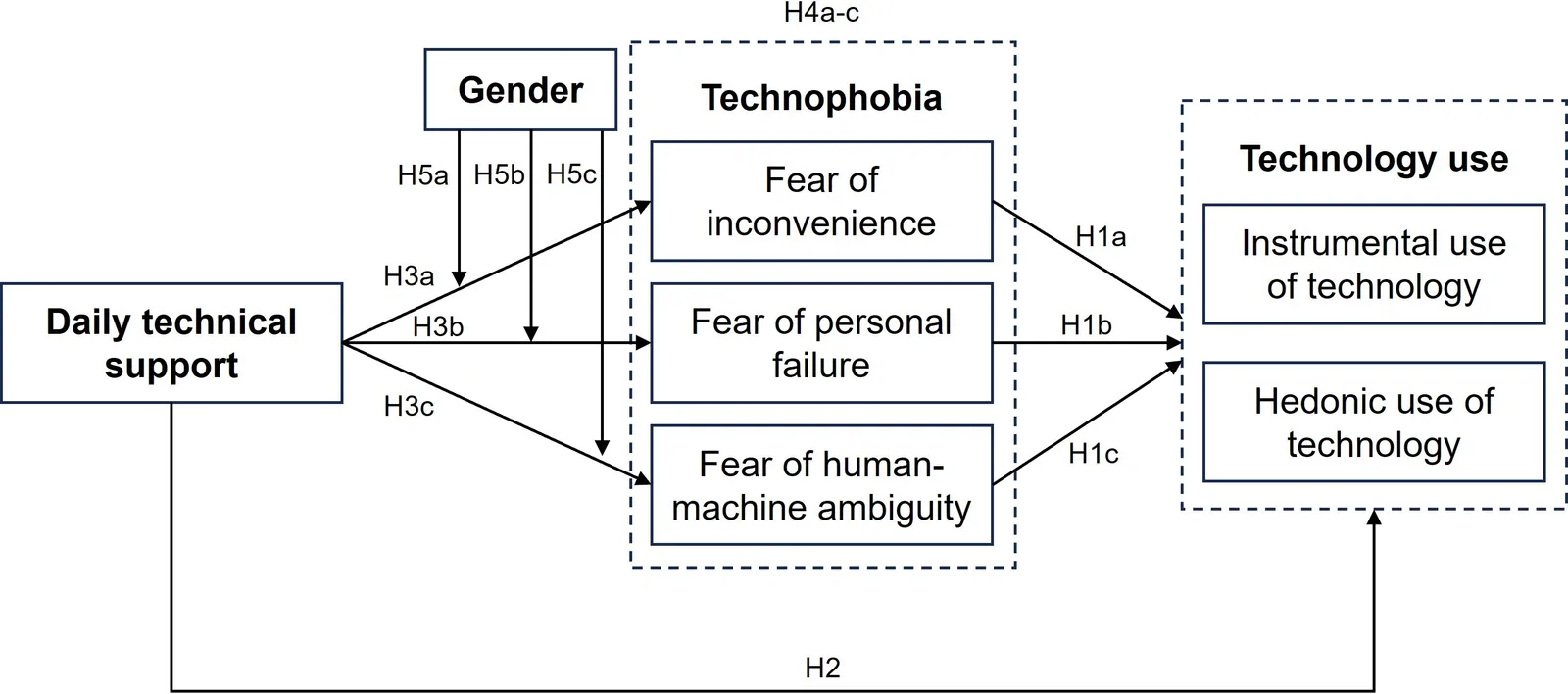
Conceptual model.

## Methods

### Sample and Procedures

We recruited Chinese adults aged 55 years and older through online platforms and offline communities in Nanjing, China. Inclusion criteria were (1) age of ≥55 years, (2) sufficient cognitive ability to complete the questionnaire independently or with assistance, and (3) the ability to understand the questionnaire content. The target sample size was determined based on recruitment feasibility during the predefined data collection period. To further evaluate whether the achieved sample size was adequate for the proposed moderated mediation analyses, we conducted a sensitivity analysis using G*Power 3.1 (Franz Faul). Because the hypothesized involved first-stage moderation, the analysis focused on the incremental variance explained by the interaction term in a linear multiple regression model (fixed model, *R*² increase). Each first-stage regression included 3 predictors and 9 covariates, resulting in a total of 12 predictors. With the final sample of 212 participants, α=.05, and power=.80, the study was capable of detecting an incremental interaction effect of approximately *f*²=.037 or larger. Thus, the achieved sample provided sufficient sensitivity to detect relatively small moderation effects in the proposed model.

A total of 230 questionnaires were collected between June and October 2024, thus yielding 212 valid responses for analysis after data screening. No missing data were present in the final dataset, as all items were set as mandatory in the online questionnaire, while trained volunteers assisted participants with questionnaire completion during offline data collection.

### Ethical Considerations

This study was approved by the Research Ethics Committee of the School of Aging Services and Management, Nanjing University of Chinese Medicine (number 20240523) and was conducted in accordance with the Declaration of Helsinki. All participants provided written informed consent before participation. No personally identifiable information was collected, thereby protecting participant privacy and confidentiality. Participants received either RMB 7 (approximately US $1) or a small gift of equivalent value as compensation for their participation.

### Variables and Measures

All measures were adapted from validated scales and translated via forward-backward translation to ensure linguistic and cultural validity for Chinese older adults. Full item wording, outer loadings, reliability and validity statistics for all constructs are reported in Table 1. DTS was assessed using 3 items with 5-point ordered category and 6-point Likert-type response formats; scores from the 6-point scale were linearly rescaled to a 5-point metric for comparability, with higher scores indicating greater DTS. Instrumental and hedonic technology use were each measured using 2 items based on the frequency of use over the past week, with responses recorded on 5-point scales and higher scores indicating more frequent use. Technophobia was measured using Sinkovics et al [19] 13-item scale with a 6-point Likert-type agreement scale. Reverse-coded items were recoded prior to analysis, with higher scores indicating greater technophobia. All constructs were modeled as latent variables in the subsequent analyses. Covariates included age, marital status, residence, education, economic status, functional status, subjective health, number of children, and social activity participation, with their coding and categories reported in Table 2. Gender was coded as 0 for men and 1 for women.

**Table 1.** Assessment of construct reliability and validity.

Construct and items	L^a^	Cronbach α	ρ_a_^b^	ρ_c_^b^	AVE^c^
DTS^d^	—^e^	.689	0.778	0.832	0.634
DTS1: with how many peers around your age do you have regular contact about how to use digital technologies?	0.916				
DTS2: with how many younger adults (aged 50 years and younger) do you have regular contact about how to use digital technologies?	0.892				
DTS3: to what extent can you get help from these people when having difficulties in using digital technology?	0.819				
TP-TI^f^	—	.759	0.761	0.837	0.508
TP-TI1: digital devices make me feel irritated.	0.784				
TP-TI2: the ubiquity of digital devices in our daily life makes me dissatisfied.	0.771				
TP-TI3: I feel comfortable when using digital devices.	0.761				
TP-TI4: digital devices make daily activities easier.	0.709				
TP-TI5: I enjoy the convenience provided by digital devices.	0.785				
TP-PF^g^	—	.929	0.933	0.946	0.779
TP-PF1: I feel some anxiety when approaching digital devices.	0.784				
TP-PF2: I believe most people know better than I do how to use digital devices.	0.671				
TP-PF3: I feel frustrated when using digital devices.	0.661				
TP-PF4: just thinking about digital devices makes me nervous.	0.706				
TP-PF5: digital devices are frightening.	0.685				
TP-HMA^h^	—	.785	0.829	0.872	0.695
TP-HMA1: I prefer to have people help me with daily activities rather than using digital devices.	0.878				
TP-HMA2: I feel more confident interacting with people than using digital services.	0.791				
TP-HMA3: our daily activities should not be solved by machines.	0.829				
IU^i^	—	.836	0.890	0.900	0.751
IU1: I used digital technologies for daily tasks (eg, information seeking and online payment).	0.916				
IU2: I used digital technologies for communication (eg, private messages, phone calls, and online communities).	0.901				
HU^j^	—	.668	0.706	0.856	0.748
HU1: I used digital technologies for individual leisure activities (eg, online games and short videos).	0.828				
HU2: I used digital technologies for group leisure activities (eg, online multiplayer games).	0.900				

a L: outer loadings.

b ρa and ρc: composite reliability.

c AVE: average variance extracted.

d DTS: daily technical support.

e Not available.

f TP-TI: technophobia-technology inconvenience. Items 3, 4, and 5 were reverse-coded.

g TP-PF: technophobia-personal failure.

h TP-HMA: technophobia-human-machine ambiguity.

i IU: instrumental use.

j HU: hedonic use.

**Table 2.** Description of participants (N=212).

Characteristics	Values, n (%)
Age (years)	
55‐59	24 (11.3)
60‐64	64 (30.2)
65‐69	48 (22.6)
70‐74	42 (19.8)
75+	34 (16.0)
Gender	
Men	67 (31.6)
Women	145 (68.4)
Marital status	
Married and/or living together with a partner	197 (92.9)
Divorced or separated	15 (7.1)
Residence	
City	203 (95.8)
Countryside or village	9 (4.2)
Economic status	
Very poor	0 (0.0)
Poor	21 (9.9)
Fair	127 (59.9)
Good	58 (27.4)
Very good	6 (2.8)
Number of children	
0	15 (7.1)
1	115 (54.2)
2	62 (29.2)
≥3	20 (9.4)
Physical function	
Greatly limits daily activities	5 (2.4)
Sometimes limits daily activities	19 (9.0)
Minimally limits daily activities	93 (43.9)
No health condition or health condition causes no limitation	95 (44.8)
Subjective health	
Excellent	20 (9.4)
Very good	42 (19.8)
Good	66 (31.1)
Fair	77 (36.3)
Poor	6 (2.8)
Education level	
Less than 12 years of education	126 (59.4)
12 years of education	31 (14.6)
Professional or vocational education	11 (5.2)
Higher education (university or college)	44 (20.8)
Social activity participation	
Yes	146 (68.9)
No	66 (31.1)
Device ownership^a^	
Smartphone	187 (88.2)
Smart TV	48 (22.6)
Smart watch or wearable device	26 (12.3)
Personal digital assistant	20 (9.4)
Laptop	4 (1.9)
Tablet	15 (7.1)
Other devices	16 (7.5)
Number of same-age technical supporters	
0	27 (12.7)
1‐2	27 (12.7)
3‐5	62 (29.2)
6‐10	41 (19.3)
>10	55 (25.9)
Number of younger-adult technical supporters	
0	31 (14.6)
1‐2	37 (17.5)
3‐5	40 (18.9)
6‐10	48 (22.6)
>10	56 (26.4)

a Percentages do not sum to 100% because participants could select more than one device type.

### Common Method Bias

Procedural and statistical controls were implemented to address potential common method bias that might arise from self-reported data. Procedurally, a pilot test with older adults improved item clarity and reduced bias. The questionnaire items were phrased in neutral language to avoid leading responses. Statistically, Harman single-factor test was performed via exploratory factor analysis with all measurement items. The results indicated that no single factor accounted for more than 50% of the total variance. Interconstruct correlation coefficients ranged from 0.154 to 0.814 with all values being below the 0.90 threshold [67]. Variance inflation factors (VIFs) for all constructs were between 1.054 and 3.261, which were below the critical value of 3.3 [68], thus eliminating severe multicollinearity.

Figure 2 illustrates the final partial least squares structural equation modeling (PLS-SEM) model with standardized path coefficients.

**Figure 2.**
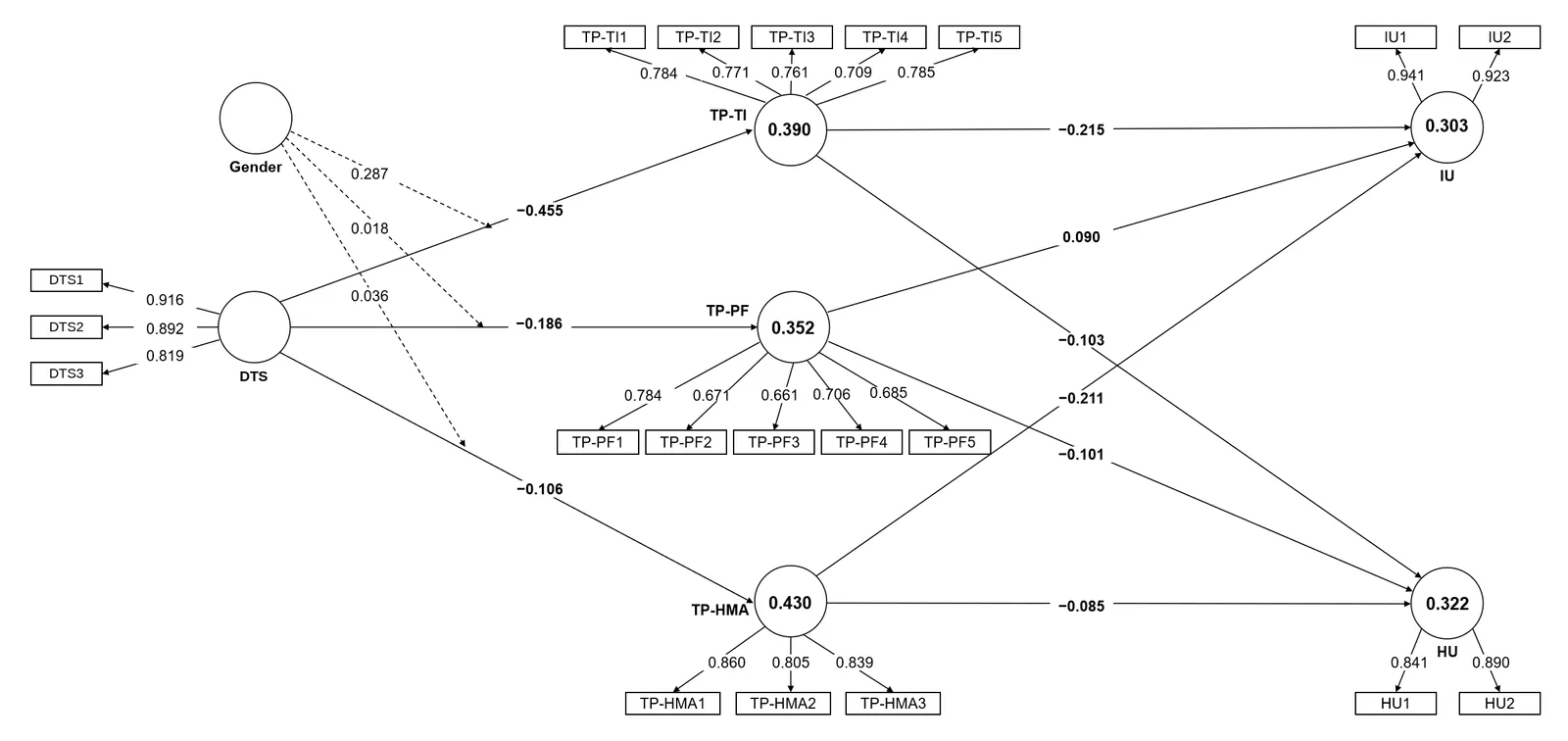
PLS-SEM results. Standardized path coefficients are shown on structural paths, and values inside endogenous constructs represent explained variance (*R*²). Outer values represent indicator loadings. Dashed lines indicate moderation effects of gender. HU: hedonic use; IU: instrumental use; DTS: daily technical support; TP-HMA: technophobia-human-machine ambiguity; TP-PF: technophobia-personal failure; TP-TI: technophobia-technology inconvenience.

### Data Analysis

PLS-SEM was conducted in SmartPLS version 4.1.1.4 (SmartPLS GmbH) to estimate the measurement and structural models examining how DTS and technophobia shape older adults’ technology engagement. PLS-SEM is well suited to this study because it accommodates complex models with multiple latent constructs, is robust to nonnormal survey data, and performs well with relatively small samples. Therefore, model parameters were first obtained using the partial least squares (PLS) algorithm. Statistical inference for all hypothesized effects—including direct paths, the moderation and indirect (mediated) effects—was then performed via a nonparametric bootstrapping procedure with 5000 resamples. For the moderated mediation hypothesis, conditional indirect effects were computed at different levels of the moderator, and their significance was evaluated using 95% bias-corrected and accelerated (BCa) bootstrap CIs. Effects were considered significant when the corresponding 95% CI did not include zero. This approach is consistent with recent applications of moderated-mediation designs examining sex or gender-differentiated pathways in health-related outcomes [69,70].

## Results

### Participants’ Demographic Profile

Participants’ demographic characteristics are reported in Table 2. The sample (N=212) had a mean age of 66.96 (SD 6.96; range 55‐86) years and was predominantly women (142/212, 68.4%). Most respondents lived in urban areas (203/212, 95.8%) and were married or cohabiting (192/212, 92.9%). In addition, 68.9% (146/212) reported having volunteer service experience, indicating active social participation among the sample. Regarding family structure, most reported having 1 or 2 children. Overall health was generally moderate, with over 40% (95/212) reporting no or minimal limitations in daily activities and most rating their health as fair or good. Educational attainment was relatively low, with nearly 60% (126/212) reporting fewer than 12 years of schooling and about one-fifth having completed higher education. The digital devices used by participants varied, with smartphones being the most commonly owned device (187/212, 88.2%), followed by smart televisions (48/212, 22.6%) and smartwatches or wearable devices (26/212, 12.3%). Regarding DTS, participants reported support from both same-age peers and younger adults, with 25.9% (55/212) reporting more than 10 same-age technical supporters and 26.4% (56/212) reporting more than 10 younger-adult technical supporters; only 12.7% (27/212) and 14.6% (31/212), respectively, reported having no such supporters.

### Measurement Model Analysis

Table 1 reports factor loadings, reliability, and convergent validity. Most outer loadings exceeded the recommended threshold of 0.708, ranging from 0.661 to 0.916, indicating adequate indicator reliability [71]. Convergent validity was supported, with Cronbach α ranging from 0.668 to 0.929, composite reliability (CR) from 0.832 to 0.946, and average variance extracted (AVE) from 0.508 to 0.779; all CR values exceeded 0.70 and AVE values exceeded 0.50 [72]. Discriminant validity was established using the Fornell–Larcker criterion and Heterotrait-Monotrait ratio (HTMT). As shown in Table 3, the square roots of AVE exceeded interconstruct correlations, and all HTMT ratios were below 0.90 [67].

**Table 3.** Assessment of discriminant validity^a^.

Fornell-Larcker criterion	DTS^b^	IU^c^	HU^d^	TP-TI^e^	TP-PF^f^	TPHA^g^
DTS	0.797^a^	0.495	0.495	−0.351	−0.260	−0.259
IU	0.495	0.867^a^	0.814	−0.351	−0.351	−0.423
HU	0.459	0.814	0.865^a^	−0.364	−0.373	−0.391
TP-TI	−0.351	−0.351	−0.364	0.712^a^	0.673	0.459
TP-PF	−0.260	−0.351	−0.373	0.673	0.852^a^	0.750
TP-HMA	−0.259	−0.423	−0.391	0.450	0.750	0.833^a^
HTMT^h^						
DTS	—^i^	0.631	0.669	0.504	0.306	0.317
IU	0.631	—	0.795	0.405	−0.390	−0.492
HU	0.669	0.795	—	0.486	0.461	0.520
TP-TI	0.504	0.405	0.486	—	0.673	0.732
TP-PF	0.306	0.390	0.461	0.673	—	0.865
TP-HMA	0.317	0.492	0.520	0.732	0.865	—

a Diagonal values represent the square root of the average variance extracted (AVE) for each construct; off-diagonal values represent interconstruct correlations. HTMT values are reported in the lower panel.

b DTS: daily technical support.

c IU: instrumental use.

d HU: hedonic use.

e TP-TI: technophobia-technology inconvenience.

f TP-PF: technophobia-personal failure.

g TP-HMA: technophobia-human-machine ambiguity.

h HTMT: Heterotrait-Monotrait ratio.

i Not available.

### Structural Model Analysis

#### Structural Model Evaluation

Before hypothesis testing, the structural model was assessed for collinearity, explanatory power, and predictive relevance (Table 4). Inner VIFs ranged from 1.009 to 3.404, indicating no serious multicollinearity concerns. The model explained 16.5% of variance in technophobia-technology inconvenience (TP-TI), 11.6% in technophobia-personal failure (TP-PF), and 10.6% in technophobia-human-machine ambiguity (TP-HMA), indicating modest levels of explained variance, and 27% of the variance in IU and 29.5% in HU, reflecting moderate explanatory power [71]. Effect size (*f*²) results showed that technical support had its largest impact on TP-TI (*f*²=0.126, small-to-moderate), with small effects on TP-PF (*f*²=0.034) and TP-HMA (*f*²=0.036), while effect sizes for instrumental and HU were negligible (all *f*²<0.03). All *Q*² values were above zero, indicating adequate predictive relevance.

**Table 4.** Structural model evaluation^a^.

Endogenous construct	*R*²	*f* ^2^	Inner VIF^b^ range	*Q*²
TP-TI^c^	0.165	DTS^d^ (0.126)	1.009‐2.956	0.092
TP-PF^e^	0.116	DTS (0.034)	1.009‐2.956	0.073
TP-HMA^f^	0.106	DTS (0.036)	1.009‐2.956	0.067
IU^g^	0.270	TI^h^ (0.029)	1.082‐3.404	0.141
HU^i^	0.295	TI (0.007)	1.082‐3.404	0.162

a VIF <5 (with most values <3.3) indicates no multicollinearity; *f*²=0.02, 0.15, and 0.35 denote small, medium, and large effect sizes; *Q*²>0 indicates predictive relevance.

b VIF: variance inflation factors.

c TP-TI: technophobia-technology inconvenience.

d DTS: daily technical support.

e TP-PF: technophobia-personal failure.

f TP-HMA: technophobia-human-machine ambiguity.

g IU: instrumental use.

h TI: technology inconvenience.

i HU: hedonic use.

#### Path Analysis

To further test the hypothesis, path analysis was conducted. As shown in Table 5, the effects of each dimension of technophobia on technology use (H1) showed that TP-TI significantly reduced IU (β=−0.215, 95% BCa CI −0.410 to ‐0.009; *P*=.045), while TP-HMA also negatively predicted IU (β=−0.211, 95% BCa CI −0.389 to 0.001; *P*=.04). By contrast, TP-PF did not significantly predict either IU or HU, as the corresponding 95% BCa CI included zero. Human-machine ambiguity was also significantly negatively associated with IU (β=−0.211, 95% BCa CI −0.389 to −0.001; *P*=.04). Next, DTS was found to exert a significantly positive direct effect on IU (β=0.104, 95% BCa CI 0.009 to 0.203*; P*=.04), but its direct path to HU was not significant, with the 95% BCa CIs including zero, which partially supports H2. In addition, results showed that DTS negatively predicted the dimension of perceived TP-TI (β=−0.455, 95% BCa CI −0.632 to −0.229; *P*<.001), while the effects of DTS on TP-PF and on TP-HMA were not significant, as both CIs crossed zero. Hence, only H3a is supported.

**Table 5.** Hypothesis testing^a^.

Hypotheses and paths	β^b^ (95% BCa^c^ CI)	*t* test^d^	*P* values	Supported
H1a				
TP-TI^e^ → IU^f^	−0.215 (−0.410 to −0.009)	2.01	.05	Yes
H1b				
TP-PF^g^ → HU^h^	−0.101 (−0.351 to 0.132)	0.82	.41	No
H1c				
TP-HMA^i^ → HU	−0.085 (−0.273 to 0.145)	0.80	.43	No
TP-HMA → IU	−0.211 (−0.389 to −0.001)	2.10	.04	Yes
H2				
DTS^j^ → IU	0.104 (0.009 to 0.203)	2.06	.04	Yes
DTS → HU	0.075 (−0.018 to 0.166)	1.58	.11	No
H3a				
DTS → TP-TI	−0.455 (−0.632 to −0.229)	5.47	.00	Yes
H3b				
DTS → TP-PF	−0.186 (−0.400 to 0.073)	1.57	.12	No
H3c				
DTS → TP-HMA	−0.106 (−0.286 to 0.075)	1.14	.26	No
H5a				
DTS × Gender→TP-TI	0.287 (0.052 to 0.518)	2.40	.02	Yes
H5b				
DTS × Gender→TP-PF	0.018 (−0.274 to 0.268)	0.13	.90	No
H5c				
DTS × Gender→TP-HMA	0.036 (−0.198 to 0.255)	0.32	.75	No

a CI values not including zero indicate statistical significance.

b β: standardized path coefficient.

c BCa: Bias-corrected and accelerated CI.

d *t* values were derived from PLS-SEM bootstrapping (5000 resamples); df is not applicable.

e TP-TI: technophobia-technology inconvenience.

f IU: instrumental use.

g TP-PF: technophobia-personal failure.

h HU: hedonic use.

i TP-HMA: technophobia-human-machine ambiguity.

j DTS: daily technical support.

Lastly, the interaction effect between DTS and gender was significant only for TP-TI (β=0.287, 95% BCa CI 0.052-0.518; *P*=.02), but not for TP-PF or TP-HMA, as the corresponding CIs included zero. We further conducted the simple slope analysis. Figure 3 presents that fear of inconvenience declined with increasing DTS for both genders. However, the decline was substantially steeper among males, indicating that daily support more effectively reduced perceived inconvenience for men (β=−0.455; *P*<.001) than for women (β=−0.168; *P*=.07). H5a is therefore supported.

**Figure 3.**
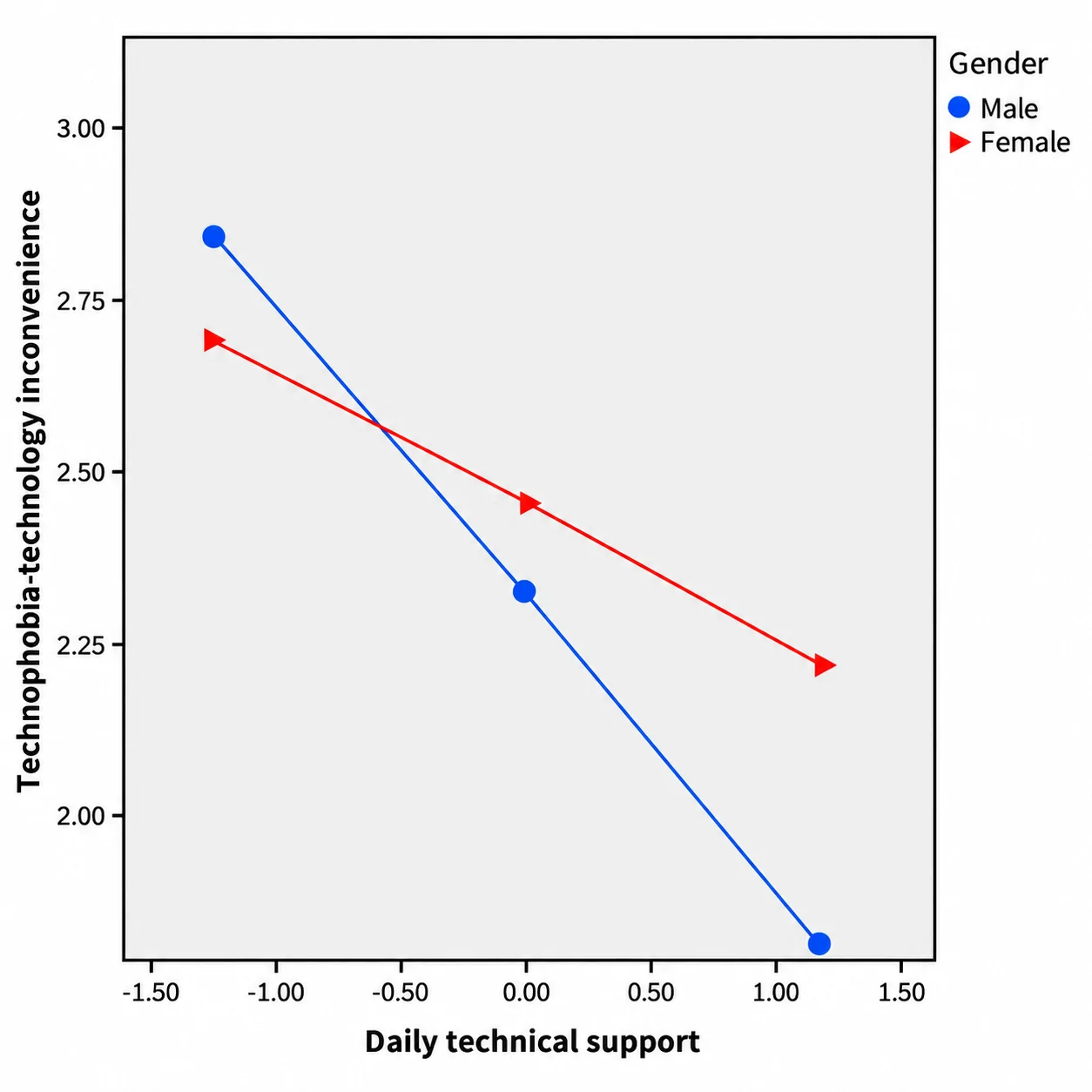
Simple slopes analysis.

Additional moderation analyses were conducted to examine whether gender moderated the overall associations between DTS and the 2 forms of technology use. Neither the interaction predicting IU (β=0.116, SE .115, *t*=1.014, 95% BCa CI −0.107 to 0.334*; P*=.31) nor that predicting HU (β=0.165, SE .114, *t*=1.444, 95% BCa CI −0.056 to 0.391; *P*=.15) was significant.

#### Moderated Mediation Effect Analysis

Furthermore, bootstrap analysis (5000 resamples, bias-corrected) was conducted to examine the moderated mediating effects of technophobia in the interaction effect between DTS and gender on 2 types of technology use (H4, H5). Gender was coded as 0=men (reference category) and 1=women (comparison category). The results indicated that only TP-TI significantly mediated the effect of DTS on IU (β=0.097, 95% BCa CI 0.020-0.204), thus suggesting that part of the positive influence of DTS on goal-oriented use operates through reducing perceptions of inconvenience. By contrast, the indirect effects through TP-PF (β=–0.017, 95% BCa CI –0.059 to 0.150) and TP-HMA (β=0.009, 95% BCa CI –0.046 to 0.146) were not supported, as the corresponding CIs included zero (Table 6). These findings indicate that among the 3 dimensions of technophobia, only TP-TI acts as a significant mediator that links DTS and instrumental technology use.

**Table 6.** Moderated mediation analysis^a^.

Hypothesis	Group	β^b^ (95% BCa CI^c^)	Supported
Overall indirect effects			
H4a			
DTS^d^ → TP-TI^e^ → IU^f^	—^g^	0.097 (0.020 to 0.204)	Yes
DTS → TP-TI → HU^h^	—	0.047 (−0.046 to 0.146)	No
H4b			
DTS → TP-PF^i^ → IU	—	−0.017 (−0.059 to 0.150)	No
DTS → TP-PF → HU	—	0.019 (−0.018 to 0.125)	No
H4c			
DTS → TP-HMA^j^ → IU	—	0.009 (−0.046 to 0.146)	No
DTS → TP-HMA → HU	—	0.022 (−0.012 to 0.091)	No
Conditional direct effects			
DTS → TP-TI	Male (0)	−0.455 (−0.657 to‐0.251)	—
DTS → TP-TI	Female (1)	−0.168 (−0.354 to 0.001)	—
DTS → TP-PF	Male (0)	−0.186 (−0.400 to 0.073)	—
DTS → TP-PF	Female (1)	−0.168 (−0.335 to ‐0.001)	—
DTS → TP-HMA	Male (0)	−0.106 (−0.286 to 0.075)	—
DTS → TP-HMA	Female (1)	−0.070 (−0.238 to 0.098)	—
Conditional indirect effects			
DTS → TP-TI → IU	Male (0)	0.098 (0.001 to 0.219)	Yes
DTS → TP-TI → IU	Female (1)	0.036 (−0.004 to 0.111)	No
DTS → TP-TI → HU	Male (0)	0.047 (−0.047 to 0.146)	No
DTS → TP-TI → HU	Female (1)	0.017 (−0.017 to 0.073)	No
DTS → TP-PF → IU	Male (0)	−0.017 (−0.074 to 0.057)	No
DTS → TP-PF → IU	Female (1)	−0.015 (−0.070 to 0.037)	No
DTS → TP-HMA → IU	Male (0)	0.022 (−0.014 to 0.089)	No
DTS → TP-HMA → IU	Female (1)	0.015 (−0.019 to 0.074)	No

a CI values not including zero indicate statistical significance. Conditional indirect effects were estimated using bias-corrected bootstrapping (5000 resamples). Gender was dummy-coded as 0=men and 1=women.

b β: Standardized path coefficient.

c BCa: Bias-corrected and accelerated CI.

d DTS: daily technical support.

e TP-TI: technophobia-technology inconvenience.

f IU: instrumental use.

g Not available.

h HU: hedonic use.

i TP-PF: technophobia-personal failure.

j TP-HMA: technophobia-human-machine ambiguity.

Conditional indirect effect analysis showed that the indirect effect of DTS on IU through TP-TI was significant for men (β=0.098, 95% BCa CI 0.001-0.219) but not significant for women (β=0.036, 95% BCa CI −0.002 to 0.116). All other indirect effects were nonsignificant for both genders, as the corresponding 95% BCa CI included zero.

## Discussion

### Principal Findings

Using a cross-sectional survey of community-dwelling older adults and PLS-SEM for hypothesis testing, the current research found that DTS lowers the technology-inconvenience dimension of technophobia, and this reduction subsequently promotes instrumental—though not hedonic—technology use, especially among older men. By contrast, DTS was not significantly related to the personal-failure or human-machine ambiguity dimensions, but higher human-machine ambiguity was indeed linked to lower IU. These results refine theory by specifying a dimension-specific and gendered mechanism, and offer practical guidance to bridge the digital divide.

### Distinct Fear Sources Linked to Instrumental Technology Use

The first finding of this research is that not all fears under the umbrella of technophobia equally predict older adults’ actual technology use. Older individuals’ instrumental (vs hedonic) use of technology was significantly influenced by their fear of inconvenience and by human-machine ambiguity but not by their fear of personal failure. Our finding is consistent with other research that has shown that extrinsic motivations, especially technology usefulness, often outweigh intrinsic motivations in determining the usage of involuntary technologies [73,74]. On the other hand, the finding from the dimension of human-machine ambiguity aligns with Smrke et al [27] findings, that older adults who are uneasy about technology’s role tend to stay away from new health technologies.

Fear of personal failure did not significantly predict either instrumental or hedonic technology use among older adults in our study. One possible explanation is that our survey captured a general, self-reported belief about technological competence rather than a contextually primed perception [75]. Research on age stereotypes suggests that the activation of negative self-stereotypes is often context dependent and more likely to occur when salient age-related cues are present [76]. As a result, older adults may not explicitly endorse negative self-evaluations in a generic survey setting, which could attenuate observed associations with actual technology use.

### The Role of DTS Among Older Adults

The key finding is that the effect of DTS on technophobia varies by dimension and gender. Daily support significantly reduced fear of inconvenience but did not significantly change fear of personal failure or human-machine ambiguity. These latter 2 dimensions are more closely related to internal psychological concerns than to the instrumental difficulties captured by fear of inconvenience. Previous research has indicated that fear of personal failure is often rooted in internalized age stereotypes about older adults’ technological incompetence [6] and typically requires the development of self-efficacy through structured learning [77]. The nonsignificant association observed in the current study differs from findings reported in some intervention studies [53,54]. One possible explanation is that older adults may respond differently to different forms of technical support [53,54]. Informal, immediate help may function primarily as instrumental problem-solving, whereas structured programs may be more capable of changing underlying confidence and motivation [74,78]. Similarly, human-machine ambiguity reflects broader attitudinal or ideological concerns about technology and its potential to replace or reduce human interaction [42]. Because TS typically targets immediate operational issues rather than these deeper beliefs, its lack of a significant association with human-machine ambiguity is understandable.

More importantly, this research uncovered that the effect of DTS on reducing the fear of inconvenience is more salient among older men than among older women. Our research resonates with existing findings, that men are more likely to address the instrumental aspect of social support than women, thus the extrinsic motivation-related fear is affected [59]. However, our research did not yield significant gender differences in the effect of technical support on fear of personal failure [63,64]. This outcome may also support the point proposed above that older adults generally seek technical support primarily to solve problems (ie, extrinsic motivation) rather than to obtain internal psychological relief (ie, intrinsic motivation). Notably, additional analyses provided no evidence that gender moderated the overall associations between DTS and either instrumental or hedonic technology use. This pattern suggests that gender may function more specifically as a boundary condition of older adults’ psychological responses to technical support rather than as a general moderator of its behavioral association with technology use.

On the other hand, DTS has a significantly positive effect on older adults’ IU of technology but no significant effect on HU. This finding is consistent with prior observations that many older adults view technology, such as mobile apps, primarily as a tool for social engagement rather than a source of entertainment [79]. Therefore, DTS, by increasing technology accessibility and decreasing frustration, demonstrates the concrete utility of technology in everyday tasks [12]. Meanwhile, many older people have well-established leisure routines outside of digital technology; even with daily assistance available, they may not be motivated to explore new entertainment technologies [79].

### Moderated Mediating Effect of Fear of Inconvenience

The gender-specific finding demonstrated that DTS boosts instrumental technology use by reducing fear of inconvenience to a greater extent among older men than among older women. This result is in line with the established TAM; that is, technical support serves as an external environmental factor that affects one’s perception toward technology per se, which is further a key driver of technology adoption [34]. It bridges 3 dimensions of technophobia and 2 classic focal predictors of technology use (ie, technology usefulness and ease of use), as well as incorporates gender as a moderator. As such, it generalizes TAM into older adults and enables a nuanced understanding of how the social environment can influence older men and women’s technology adoption behavior differently.

### Implication and Limitation

Our findings suggest a shift in how interventions to promote technology use among older adults are designed. Older adults are often stereotyped as technophobic and lacking confidence in their ability to use technology, and interventions have therefore tended to emphasize intrinsic empowerment (skills training and confidence building). Our findings indicate that more instrumental forms of support may also be important, particularly for technologies that have become difficult to avoid in everyday life. By reducing the inconvenience, effort, and practical burden associated with technology use, DTS may help older adults recognize the functional value of technology and facilitate IU. Accordingly, interventions could place greater emphasis on readily accessible, task-oriented assistance, such as just-in-time support, simplified workflows, default configurations, and age-friendly interface design with larger fonts and icons, simplified navigation, and fewer steps required to complete common tasks [80].

The stronger indirect association observed among men further suggests that older adults may not benefit from the same form of technical support to the same extent. Rather than treating gender itself as the basis for intervention, this finding highlights the importance of identifying the specific barriers that users experience and tailoring support accordingly. For individuals whose technology use is particularly constrained by perceived inconvenience, immediate and task-focused assistance may be especially effective, whereas those whose difficulties stem from other sources of technophobia may require different forms of support, such as structured learning, confidence-building, or greater reassurance about human-technology interaction. Gender may therefore serve as 1 contextual indicator of heterogeneity in how technical support is experienced, but intervention decisions should ultimately be guided by users’ specific needs and sources of technological fear [22]. Such a barrier-specific approach may provide a more precise and sustainable way to promote technology engagement among older adults.

Despite these findings, our research is not without limitations. First, the survey sample was small and skewed toward urban older adults, thus limiting the generalizability of our findings to diverse socioeconomic and rural populations. As the sample was drawn from one country, the magnitude and relative salience of these associations may also vary across cultural and social settings. Second, the cross-sectional design precludes strong causal inference about the proposed relationship between variables. Third, measures relied on self-reports, which are susceptible to recall and social desirability biases. For instance, older men may underreport technophobia because of gendered norms about technological competence. Future research should use larger and more diverse samples, including cross-cultural comparisons, and longitudinal designs. Complementing surveys with objective behavioral indicators, such as smartphone or app usage logs and passively sensed interaction traces, could also provide fine-grained evidence on how daily support translates into reduced technophobia and sustained technology use. Future studies could further examine how emerging forms of online and real-time technical support can translate these findings into practice. As older adults increasingly engage with digital technologies as everyday users, platforms could potentially use user profiles and interaction data to provide more timely and tailored support, including gender-sensitive prompts or assistance. Finally, we failed to find a significant predictor of older adults’ HU of technology; future research can focus on this specific technology domain.

### Conclusion

This study examined how DTS relates to older adults’ instrumental and hedonic technology use, with fear-specific technophobia as a mediating mechanism and gender as a moderator. TA was associated with lower fear of inconvenience, which in turn was associated with greater instrumental technology use, and this indirect effect was stronger among men than women. No comparable pathway was found for HU. Theoretically, these findings advance a more differentiated understanding of technophobia by highlighting the importance of examining specific sources of technological fear rather than treating technophobia as a unitary barrier. The absence of a comparable pathway for HU further suggests that different forms of technology use may be shaped by distinct psychological mechanisms and therefore warrant more use-specific investigation. Practically, the findings underscore the value of moving beyond one-size-fits-all approaches to digital inclusion by matching support strategies to the particular barriers older adults experience. Efforts to reduce technophobia should therefore complement generic skills training with targeted assistance that addresses specific sources of fear, practical barriers that can be alleviated through daily support, and differences in how older adults psychologically respond to such support. Such a more targeted approach may help improve the effectiveness of interventions designed to promote sustained and meaningful technology engagement in later life.

## Abbreviations

AVE: average variance extracted
BCa: bias-corrected and accelerated
CR: composite reliability
DTS: daily technical support
HTMT: heterotrait-monotrait ratio
HU: hedonic use
ICT: information and communication technology
IU: instrumental use
PLS: partial least squares
PLS-SEM: partial least squares structural equation modeling
TAM: Technology Acceptance Model
TP-HMA: technophobia-human-machine ambiguity
TP-PF: technophobia-personal failure
TP-TI: technophobia-technology inconvenience
VIF: variance inflation factor
